# Resistance profile of *Staphylococcus aureus* strains isolated from patients treated in a tertiary care hospital in Romania

**DOI:** 10.1186/1471-2334-14-S7-P59

**Published:** 2014-10-15

**Authors:** Oana Săndulescu, Andrei Grigoraş, Anca Streinu-Cercel, Ioana Berciu, Alina Cristina Neguț, Adrian Streinu-Cercel

**Affiliations:** 1 Carol Davila University of Medicine and Pharmacy, Bucharest, Romania; 2 National Institute for Infectious Diseases "Prof. Dr. Matei Balş", Bucharest, Romania

## Background

The prevalence of colonization with *Staphylococcus aureus* has been shown to increase worldwide [1,2], along with the rate of antibiotic use in medicine as well as the livestock industry. Therefore, our microbiota may be exposed to selective pressure and may develop resistance to current drugs [3].

## Methods

We have performed a study to assess the antimicrobial susceptibility profile of *S. aureus* strains isolated from clinically apparent infections treated in the National Institute for Infectious Diseases "Prof. Dr. Matei Balş". Bacterial identification and antimicrobial sensitivity testing were performed on MALDI-TOF or VITEK (bioMérieux, Paris, France).

## Results

We examined 149 strains of *S. aureus*, 44.9% from cutaneous wound infections, 31.2% from blood cultures, 8.4% from sputum samples and 15.3% from other infection sites. Of the total number of strains identified, 55.7% were resistant to methicillin, 35.1% were resistant to clindamycin (D-test results are presented separately), 30.9% were resistant to levofloxacin, 18.7% were resistant to rifampin and smaller percentages were identified for resistance to other drugs. Surprisingly, resistance was also identified to drugs that are not used in clinical practice in Romania, such as daptomycin (6.8%) or fusidic acid (4.8%), suggesting a possible international circulation of *S. aureus* strains, probably through means of nasal or axillary carriage. We excluded a mechanism of daptomycin non-susceptibility through thickened bacterial cell walls in strains of vancomycin-intermediate *S. aureus*, as all strains were susceptible to vancomycin.

## Conclusion

Antimicrobial resistance remains a major issue in clinical practice, but studies describing the local antimicrobial susceptibility patterns of important pathogens can aid in guiding first-line antibiotherapy.
